# Characterization of novel *Annona reticulata* fiber as a potential reinforcement for composite applications

**DOI:** 10.1038/s41598-025-15601-9

**Published:** 2025-08-23

**Authors:** S. Hossain, M. A. Saeed, T. Islam, S. Islam, M. A. Jalil, M. M. Rahman, S. C. Das

**Affiliations:** 1 Department of Environmental Science, BGMEA University of Fashion and Technology, Dhaka, 1230 Bangladesh; 2 Department of Natural Sciences, BGMEA University of Fashion and Technology, Dhaka, 1230 Bangladesh; 3 Department of Textiles, Merchandising, and Interiors, University of Georgia, Athens, GA 30602 USA; 4 Department of Textile Engineering, Jashore University of Science and Technology, Jashore, 7408 Bangladesh; 5 Department of Textile Engineering, BGMEA University of Fashion and Technology, Dhaka, 1230 Bangladesh; 6 Pulp and Paper Research Division, Bangladesh Council of Scientific and Industrial Research, Dhaka, 1205 Bangladesh; 7 Department of Manufacturing and Civil Engineering, Norwegian University of Science and Technology, Gjøvik, 2815 Norway

**Keywords:** *Annona reticulata L.*, Cellulosic fibers, Natural fibers, Sustainable materials, Fibers characterization, New reinforcement for composites, Engineering, Materials science

## Abstract

With the rising demand for sustainable and environmentally friendly materials, there is growing interest in alternative natural fibers for potential biocomposite applications. This study introduces a novel source of lignocellulosic fibers derived from the bark of *Annona reticulata* L., an abundant and underutilized bioresource. This novel fiber aims to meet the growing demand for eco-conscious textiles and biocomposite materials by preserving biodegradability and minimizing environmental impact. The fibers were extracted using a simple, chemical-free, short-duration water retting process, followed by drying. The dried fibers were characterized via chemical composition analysis, FTIR spectroscopy, density measurement, XRD, thermal analysis, morphological examination, and tensile testing. It was revealed that the fiber contains 56.26, 17.56, and 16.74% cellulose, hemicellulose, and lignin, respectively, while the density was found 1.33 g/cm^3^. XRD analysis indicates a crystalline index of 65% and a crystalline size of 3.34 nm, suggesting a moderately ordered cellulose structure. Thermogravimetric analysis confirmed thermal stability up to 258 °C, with maximum degradation occurring at 373 °C. FTIR analysis confirmed the presence of characteristic functional groups, including O–H stretching at 3309 cm⁻¹ and C–H stretching at 2928 cm⁻¹ in cellulose, as well as C = C stretching at 1514 cm⁻¹ in lignin, typical of lignocellulosic fibers. SEM imaging further revealed a fibrillar and bundled microstructure. Tensile testing showed an average tensile strength of 327 MPa and a Young’s modulus of 4.3 GPa, supporting the potential of *Annona reticulata* fibers for load-bearing applications. The findings of this study are comparable to those of commonly employed and some other new lignocellulosic fibers exhibited the potential of *Annona reticulata* for bicomposite applications.

## Introduction

Plant-based natural fiber-reinforced composites offer numerous advantages over traditional synthetic fiber-reinforced composites (GFRPs or CFRPs), including lightweight construction, enhanced specific stiffness, biodegradability, low costs, reduced energy requirements, ease of fabrication, and minimal tool wear. These benefits are driving a significant shift in materials research toward environmentally responsible alternatives^1–3^. Consequently, plant-based lignocellulosic fiber composites are now widely used across various sectors, including construction, automotive manufacturing, sports equipment, recreational and domestic appliances, where both performance and sustainability are key priorities^2,4^. These composites also show promise in advanced applications such as aerospace, wind energy, and space technologies^2,4^.

In recent years, the scientific community has increasingly embraced natural fibers across diverse fields due to their lightweight nature, broad availability, and excellent insulating properties. As renewable resources, natural fibers present a viable alternative to synthetic fibers in composite applications^5^. Over the past two decades, significant advancements have been made in developing natural fiber-based and eco-friendly composites, driven by growing environmental awareness and concerns over the adverse health and ecological impacts of synthetic fibers. Prolonged exposure to synthetic fibers has been linked to serious health issues, including cancers and skin disorders^6^. Furthermore, synthetic fibers are non-biodegradable, offer limited recycling potential, and are expensive or troublesome to some extent, and contribute significantly to ecological imbalance^3,7^.

The development of biocomposites depends on the effective extraction and utilization of lignocellulosic fibers derived from plant sources, which are primarily composed of cellulose, hemicellulose, lignin, pectin, and waxes^8^. These constituents directly influence fiber morphology, thermal behavior, and mechanical performance. Additionally, factors such as the plant species, the specific plant part used (leaf, root, or stem), extraction technique, maturity stage, and environmental growth conditions significantly affect the resulting fiber’s chemical composition and crystallinity, thereby impacting its applicability in composite systems^2,9–11^.

However, natural fibers often contain hydrophobic contaminants such as waxes, oils, pectin, dust, or surface cracks, which hinder adequate fiber–matrix adhesion and compromise mechanical performance. To overcome these issues, chemical treatments such as alkalization, acetylation, and silane treatment have been employed to enhance the hydrophobicity of natural fibers. Alkali treatment effectively removes surface impurities and waxy substances, eliminates weak amorphous regions, and reduces hydrophobicity. This enhances fibrillation and increases surface roughness, improving bonding with the matrix^12^. Silane treatment, on the other hand, facilitates the formation of strong chemical bonds through siloxane (-Si-O-C-) linkages, standardizing the fiber surface and promoting dispersion within hydrophobic matrices. As a result, silane modification significantly enhances tensile strength, flexural strength, and modulus properties of the resulting composites^13,14^.

Among various plant sources, stem-derived fibers - such as jute, flax, hemp, ramie, kenaf, okra, artichoke, *Grewia tilifolia*, and *Prosopis juliflora* – exhibit superior mechanical properties due to their greater length and thicker cell walls^15^. Their performance is largely attributed to high cellulose content, a linear polysaccharide composed of glucose monomers, which correlates with improved tensile strength and thermal stability. Factors such as low spiral angles of cellulose microfibrils, reduced fiber diameter, and increased fiber length further contribute to improved mechanical performance^2,16^.

Surface morphology also plays a crucial role in the interfacial bonding between fibers and polymer matrices. According to Uddin et al.^17^, a rough fiber surface enhances matrix adhesion, whereas smooth surfaces may result in weak interfacial bonding. Indran et al.^18^ demonstrated that *Cissus quadrangularis* root fibers possess a lower density than conventional E-glass fibers, yet exhibit comparable mechanical strength, highlighting their suitability for both textile and structural applications. Similarly, Hossain et al.^19,20^ found that fibers extracted from jack tree stems exhibit characteristics on par with established commercial natural fibers, reinforcing the importance of exploring underutilized plant resources.

Although synthetic fibers still continue to offer higher strength, the escalating demand for biodegradable and sustainable alternatives has intensified research into natural fiber-reinforced composites^21^. Their environmentally friendly profile, combined with functional properties such as a high specific stiffness, makes them attractive for future materials innovation^3^. For example, Arthanarieswaran et al.^22^ explored *Acacia leucophloea* bark fibers as reinforcement in structural applications, while Hyness et al.^23^ underscored the importance of high cellulose content and crystallinity index in determining fiber reinforcement efficiency.

In this context, identifying and characterizing new and underexplored plant fibers is essential for advancing sustainable materials research. One such plant is *Annona reticulate* L., widely distributed across tropical and subtropical regions. Native to the Americas - particularly the West Indies and South America - it is now extensively cultivated in countries such as Bangladesh, India, Pakistan, Malaysia, Cuba, Colombia, Australia, Brazil, various parts of Africa, Taiwan, and others^24^. It is mainly cultivated for its fleshy, sweet, pleasant, and nutritious fruit. In some regions, *Annona reticulata* is also regarded as a weed, and naturally it is spreading to other areas^25^. This small tree features smooth branches and numerous lateral shoots, and its bark is known to contain high levels of tannins, alkaloids, and phenolic compounds^24^. It is commonly found in home gardens, wild areas, and along roadsides in Bangladesh. Its widespread prevalence is primarily attributed to its fast growth and exceptional adaptability to diverse environmental conditions. These characteristics make it a promising source of raw material, as it can rapidly produce large reserves of biomass.

Compared to other plants, *Annona reticulata* L. offers distinct advantages for fiber extraction, including abundant availability, good yield, ease of processing without specialized equipment, and a short retting duration of 10–14 days. Notably, it also enables the extraction of long bundle fibers suitable for both unidirectional and multidirectional reinforcement mats. The integration of new, abundantly available natural fibers with acceptable material properties can play a vital role in enhancing composite production, particularly in developing countries, thereby addressing a portion of global material demand. To the best of our knowledge, no prior studies have systematically investigated the extraction and characterization of fibers from *Annona reticulata* L. bark, leaving a significant research gap. The primary objective of this study is to extract and investigate the physico-chemical, thermal, morphological and tensile properties of the fibers to assess their potential as a new reinforcement material for the biocomposite applications.

## Experimental

### Fiber extraction

Fresh and mature branches of *Annona reticulata* L. were collected from the Rajshahi region of northern Bangladesh. The bark was carefully peeled from the branches and subjected to water retting by immersing it in clean water for 10–14 days, allowing microbial degradation of pectins and hemicelluloses. Following the retting process, the fibers were manually separated from the bark, thoroughly washed with distilled water to remove residual impurities, and air-dried under ambient conditions for seven days.

The extracted fibers were then stored in sealed containers at room temperature and used for further analysis without any additional chemical treatment, in order to preserve their native structural integrity.

**Fig. 1:**
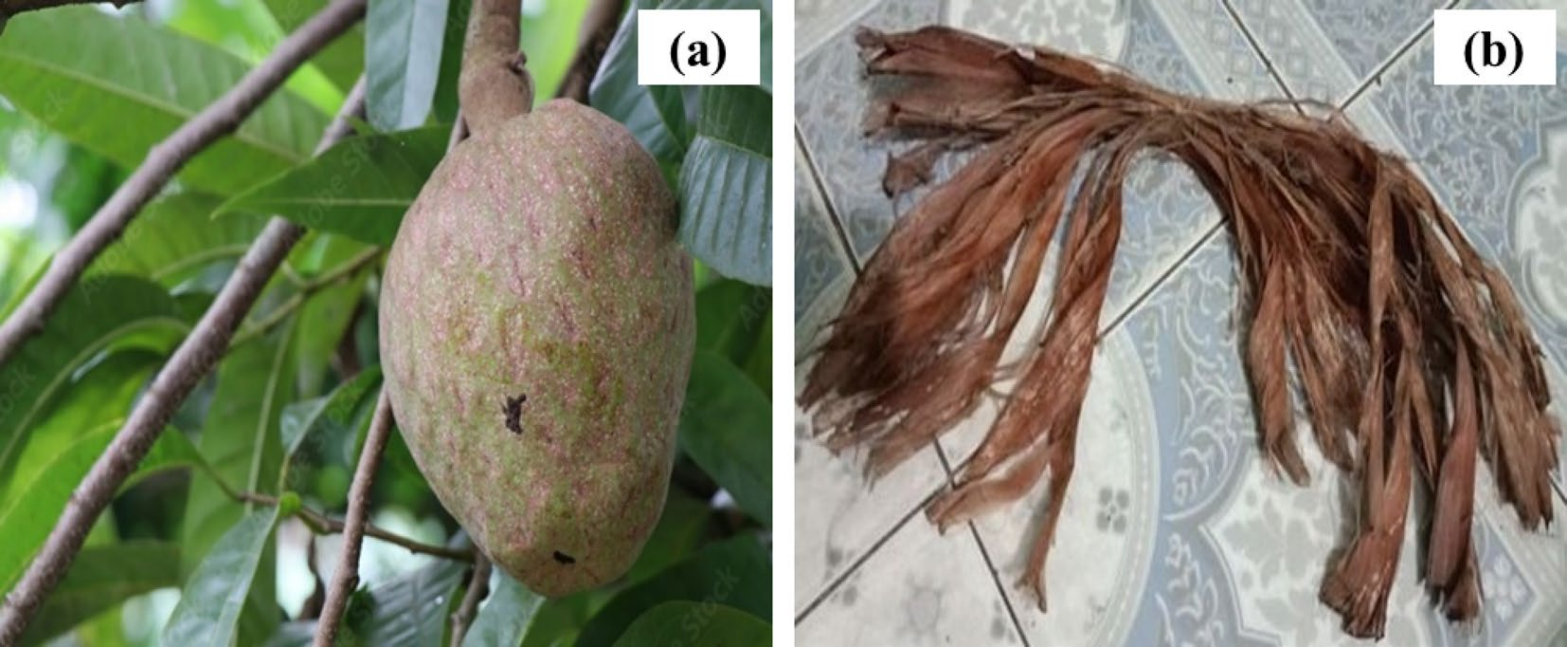
Images of **(a) **fresh branches of *Annona reticulata* L. and **(b)** extracted bast fibers.

Figure 1  illustrates the fresh branches of *Annona reticulata *L. plant (a) and the extracted fibers (b).

### Chemical composition analysis

The chemical composition of the extracted *Annona reticulata* L. fibers was analyzed by following the procedures according to TAPPI (Technical Association of the Pulp and Paper Industry) standardize methodology^19,26^. Soxhlet extraction was performed with a 1:2 (v/v) ethanol–toluene mixture in accordance with TAPPI T204 om-88 to determine the extractive content. Holocellulose was isolated by treating the extractive-free fibers with sodium chlorite (NaClO_2_), followed by extraction with sodium hydroxide (NaOH) to obtain α-cellulose (TAPPI T203 om-93). The residual lignin content was measured using the Klason method (TAPPI T222 om-88), while ash content was determined according to TAPPI T211 om-93^19,26^.

### FTIR spectroscopy

FTIR (Fourier transform Infrared) spectroscopy analysis was conducted to identify the functional groups present in the raw fibers. The spectra were recorded using an IR Prestige 21 spectrophotometer (Shimadzu, Japan) over the range of 4000–500 cm⁻¹, with a resolution of 4 cm⁻¹. The spectral data were processed using OriginPro 2025b software *(details in* Sect. 2.9*)*, and peak assignments were made based on literature data^27^.

### Fiber density measurement

The density of the raw fiber was determined by the liquid displacement method using distilled water and a pycnometer^19,28^. The density was calculated using the Eq. (1):

1 $$\:{\rho\:}_{f}=\frac{({m}_{2}-{m}_{1})}{({m}_{3}-{m}_{1})-({m}_{4}-{m}_{2})}{\rho\:}_{w}$$

Where:

*ρ*_*f*_ = Fiber density (g/cm^2^).

*ρ*_*w*_ ​= Water density (0.997 g/cm^2^ at 25 °C).

m_1_ = Mass of the empty pycnometer.

m_2_ = Mass of the pycnometer with fiber.

m_3_​ = Mass of the water-filled pycnometer.

m_4_ = Mass of the pycnometer with both fiber and water.

### X-ray diffraction (XRD) analysis

The crystalline properties of the *Annona reticulata* fiber were evaluated using an X-ray diffractometer (Smart Lab SE, Rigaku, Japan). The instrument operated at a tube current of 50 mA and voltage of 40 kV, using CuKα radiation (λ = 1.54 Å). Fiber samples were ground and pressed into pellets prior to analysis. The diffraction patterns were recorded over a 2θ range of 10° to 50°, with a scan rate of 15°/min and a step size of 0.01°. The crystallinity index (CI) was calculated using the Segal empirical method, Eq. (2)^29^:

2 $$\:\text{C}\text{I}\%=\frac{{I}_{200}-{I}_{am}}{{I}_{200}}\times\:100$$

where *I*_*200*_ is the maximum intensity of the diffraction peak at approximately 2θ = 22.6° (crystalline region), and *I*_*am*_ ​ is the minimum intensity between the peaks, typically at around 2θ = 18.9° (amorphous region).

The crystallite size (CS) was determined using the Scherrer Eq. (3):

3 $$\:\text{C}\text{S}=\frac{K\lambda\:}{\beta\:cos\theta\:}$$

where:

*K* = 0.89 is the Scherrer’s constant, *β* is the peak’s full width at half maximum in radians, *λ* is the wavelength of radiation and *θ* is the Bragg angle corresponding to half of the 2*θ* angle.

### Thermal analysis

Thermal stability and decomposition behavior of the fibers were analyzed using a thermogravimetric analyzer (TGA; Shimadzu DTG-60 H) equipped with simultaneous thermogravimetric and differential thermal analysis (TG-DTA) capabilities. The analysis was conducted under a N_2_ atmosphere to prevent oxidation, with a heating rate of 10 °C/min from room temperature up to 600 °C^30^.

### Morphological analysis

The surface morphology of the fibers was examined using a field emission scanning electron microscope (FEG-SEM; Zeiss Sigma 300) operating at 15 kV. Before SEM investigation, the *Annona reticulata* fiber specimens were sputter-coated with a 10 nm thin layer of gold using a Quorum Q150R S Plus sputter coater to enhance conductivity and image quality. A 3D surface topology map was generated using Gaussian software to visualize the microstructural features in greater detail.

### Tensile strength analysis

The tensile properties of the extracted *Annona reticulata* fibers were evaluated using a universal testing machine (Instron 3343, maximum load capacity of 1.0 kN) in accordance with ASTM D3822/D3822M-14(2020) standards. The test was performed at a crosshead speed of 3 mm/min with a gauge length of 80 mm. Prior to testing, each fiber was conditioned under standard laboratory conditions (65% RH and 21 ± 1 °C) for 24 h. Five replicates were tested to ensure statistical reliability. The average tensile strength, elongation at break, and Young’s modulus were calculated and analyzed.

### Software

OriginPro 2025b (Version 2025b, OriginLab Corporation, Northampton, MA, USA https://www.originlab.com/Newst) was used to analyse the raw data, perform statistical analysis and generate graphs or plots for this study.

### Plant material declaration

The *Annona reticulata* L. bark used in this study was collected from naturally grown sources in the Rajshahi district of Bangladesh. It is a widely available plant in Bangladesh and other tropical and subtropical countries. The plant (*A. reticulata* L.) is not listed as endangered or protected, and no special permits were required for its collection. The use of this plant material complied with all relevant institutional, national, and international guidelines and legislation.

### Identification statement

The plant species was taxonomically identified by Mr. Khandakar Kamrul Islam, Senior Scientific Officer at the Bangladesh National Herbarium, Ministry of Environment, Forest and Climate Change, Mirpur-1, Dhaka 1216, Bangladesh. A voucher specimen has been deposited at the Herbarium under the accession number **DACB 135364**.

### Plant use statement

The plant (*Annona reticulata L.*) used in this study is naturally grown in Bangladesh. No permission was required to collect and use the plant, and no protected or endangered species were involved.

## Results and discussion

### Fiber extraction

In this study, *Annona reticulata* bark fibers were extracted using an eco-friendly method involving immersion in fresh water. This water retting process facilitates fiber extraction by softening and dissolving unwanted substances in the bark. The microbial degradation of non-fibrous tissues was monitored daily through the gradual softening of bark and the increasing visibility of fibrous components. After 10–14 days of retting, the fibers were manually separated—a process that is simple, cost-effective, and sustainable.

Various methods are available for extracting fibers from cellulosic sources, including water retting, mechanical processing, and chemical treatments. Among these, water retting is widely regarded as the most practical, productive, and environmentally friendly technique. However, this natural process is not suitable for all types of fiber extraction. In contrast, chemical treatments, although effective, are hazardous, costly, and corrosive to processing equipment^19,31,32^.

### Chemical composition

Table 1 shows the chemical composition of *Annona reticulata* bark fiber. It was found that the fiber comprises 56.26% cellulose, 17.56% hemicellulose, 16.74% lignin, 8.92% extractives, 0.32% ash, and a moisture content of 10.47%. These values are further contextualized against other lignocellulosic fibers and agricultural residues in Table 2.

A key finding is the relatively high cellulose content (56.26%), which positions *Annona reticulata* bark fiber as a promising source of cellulosic biomass. This cellulose level surpasses that of commonly used fibers such as *Acacia caesia* (54.08%), coir (32–43.8%), and kenaf (31–72), and is comparable to *Carica papaya* bark (58.71%) and areca palm-based biomass (57.35–58.21%). High cellulose content is typically associated with enhanced mechanical strength and thermal stability, making the fiber suitable for applications in composite materials^33^, textile materials^34^ or can be a viable biosource for the nanocellulose generations^35,36^. The hemicellulose content (17.56%) also plays a significant role, as it influences moisture sensitivity, biodegradability, and thermal degradation behavior^37,38^.

Compared to other fibers, *Annona reticulata* exhibits a moderate hemicellulose level — higher than that of sisal (10-14.2%), coir (0.15-20%), and *Aristida hystrix (11.35%)*, but lower than banana (10–19%) and *Acacia caesia (21.52%)*. This intermediate level supports controlled biodegradation and favorable thermal transitions, which are critical for green material applications.

In terms of lignin, *Annona reticulata* contains 16.74%, a moderate amount that contributes to fiber rigidity, hydrophobicity, and resistance to microbial degradation^39^. This lignin content is higher than that of most bast fibers such as jute (11.8–13%), flax (2–5%), sisal (8–14%), hemp (3.7–10%) and banana (5%), yet lower than that of coir (40–45%), Areca fruit Husk (23.17–24.16%), and *Acacia caesia (18.14%).* This balance suggests a favorable profile for both mechanical reinforcement and functional biodegradability in biocomposite applications.

**Table 1:** Chemical composition of *Annona reticulata* bark fiber.

Cellulose (%)	Hemicelluloses (%)	Lignin (%)	Extractives (%)	Ash (%)	Moisture content/regain (%)
56.26	17.56	16.74	8.92	0.32	10.47 / 11.70

**Table 2:** Comparative chemical composition of *Annona reticulata* and other plant fibers.

Fiber	Cellulose (%)	Hemicellulose (%)	Lignin (%)	References
*Annona reticulata* bark	56.26	17.56	16.74	This study
*Carica papaya* bark	58.71	11.8	14.26	^40^
Coir	32–43.8	0.15–20	40–45	^8^
Kenaf	31–72	20.3–21.5	8–19	^8^
*Acacia caesia*	54.08	21.52	18.14	^41^
*Aristida hystrix*	59.54	11.35	8.42	^42^
Jute	59–71.5	13.6–20.4	11.8–13	^8^
Areca fruit Husk	57.35–58.21	13–15.42	23.17–24.16	^43^
Areca palm leaf stalk	57.49	18.34	7.26	^44^
Banana	63–67.6	10–19	5	^8^
Sisal	66–78	10–14.2	8–14	^8^
Jack tree fiber	79.32	8.01	6.77	^19^
Flax	62–72	18.6–20.6	2–5	^8^
Hemp	67–74.4	15–22.4	3.7–10	^8^

### FTIR analysis

**Fig. 2:**
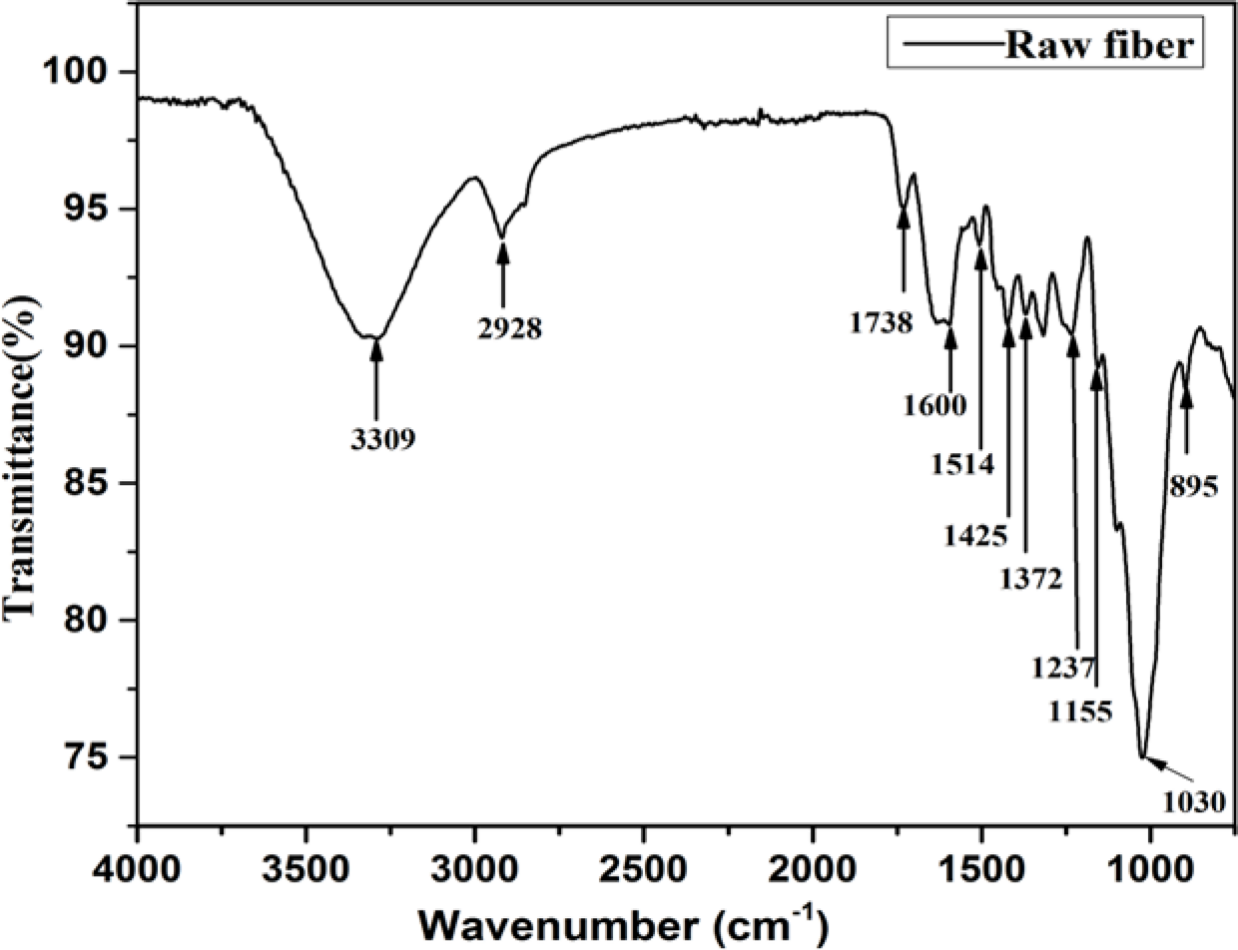
FTIR Spectra of *Annona reticulata* fiber.

The FTIR spectra of *Annona reticulata* fiber is presented in Fig. 2. The broad peak at 3309 cm⁻^1^ corresponds to –OH stretching, indicating -OH groups characteristic of cellulose and hemicellulose^36^. The absorption at 2928 cm⁻^1^ confirms the presence of C-H stretching in CH₂ and CH₃ groups, supporting the aliphatic nature of the polysaccharides^36,45^. The peak at 1738 cm⁻^1^ is attributed to C = O stretching from ester linkages in hemicellulose and lignin derivatives such as ferulic and p-coumaric acids^36,46^. Weak peaks at 1600 and 1514 cm⁻^1^ reflect the vibrations of aromatic rings and absorbed moisture in the cellulose matrix (lignin)^36,45,47^. The signals at 1425 and 1372 cm⁻^1^ indicate CH₂ bending and pyranose ring vibrations of cellulose^45,47^. Decreased intensity at 1237 and 1155 cm⁻¹ suggests the presence of lignin acetyl groups and holocellulose bridge structures^36^. Strong peaks at 1030 and 895 cm⁻^1^ correlate with C–O stretching and CH₂ rocking, confirming β-(1→4) glycosidic linkages in the glucopyranose units of cellulose and hemicellulose^36,45^. Thus, the FTIR analysis validates the presence of all key lignocellulosic components, supporting the chemical composition data. The summary of functional groups corresponding to different wavenumbers identified during FTIR analysis are presented in Table 3.

**Table 3:** Summary of functional groups in FT-IR spectra.

Wavenumber (cm^− 1^)	Vibration	Source	References
3309	O–H stretching	Polysaccharide	^36^
2928	C–H stretching	Cellulose, Hemicellulose	^45^
1738	C = O stretching	Hemicellulose	^46^
1600	O–H in H_2_O, Bending	Moisture	^45,47^
1514	C = C aromatic symmetrical stretching	Lignin	^36,45,47,48^
1425	CH_2_ symmetric bending	Cellulose, lignin	^45,47,48^
1372	C–H bending	Cellulose	^45,47,48^
1237	C–O stretching	Lignin	^65,36^
1155	C–O–C anti-symmetrical stretching	Lignin	^65,36^
1030	C–O stretching	Cellulose	^36,45^
895	Glycosidic bonds symmetric ring stretching	Polysaccharide	^36,45^

### X-Ray diffraction (XRD) analysis

**Fig. 3:**
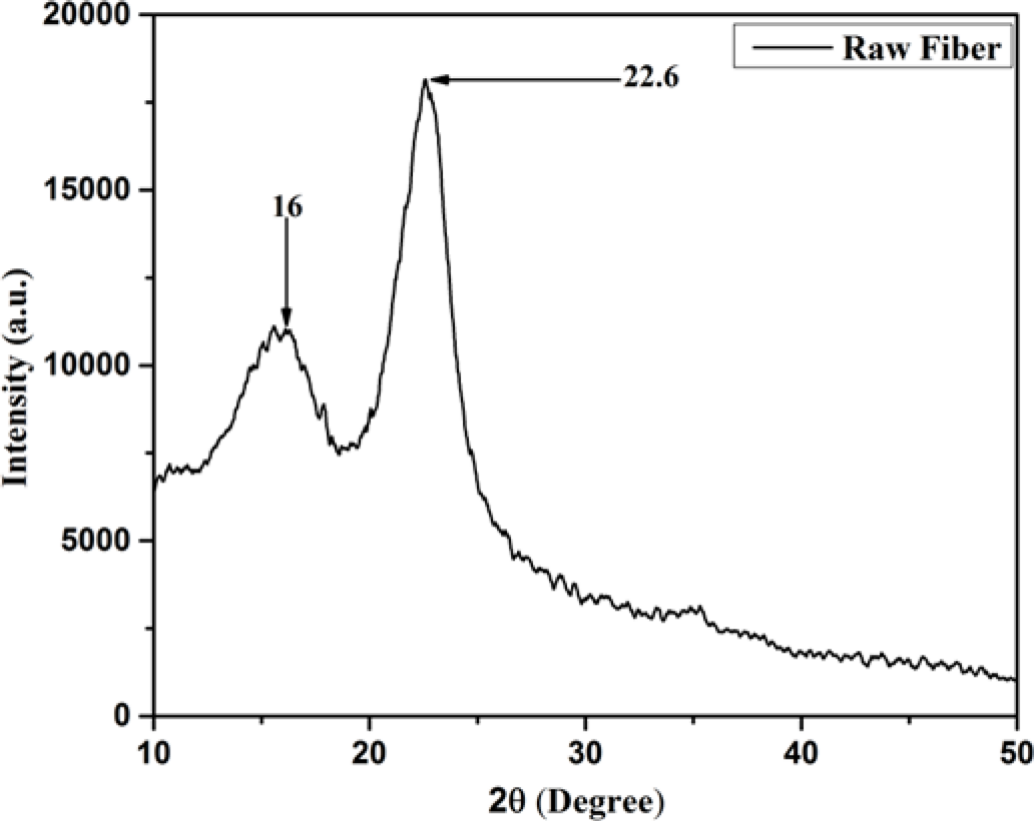
X-ray diffraction patterns of *Annona reticulata* fiber.

The XRD pattern of raw *Annona reticulata* fiber as shown in Fig. 3, reveals a predominantly semi-crystalline structure, as evidenced by the flat diffraction profile^49^. It is seen from Fig. 3, two distinct peaks are observed at ~ 16° (0 1 1) and ~ 22.6° (0 0 2), which are characteristic of plant-based lignocellulosic fibers^16,50^. These peaks correspond to the crystalline regions of cellulose I_β_, indicating the coexistence of both crystalline and amorphous phases in the fibers^50^. The peak at ~ 16° is also associated with residual non-cellulosic components, such as hemicellulose and lignin, further confirming the partially untreated nature of the raw *Annona reticulata* fiber^16,50^.

The calculated crystallinity index of 65% possesses a moderately high crystalline structure in *Annona reticulata* fiber, which is beneficial for enhancing mechanical strength and dimensional stability in composite applications. The crystallite size, determined to be 3.34 nm, reflects a reasonably ordered arrangement of cellulose chains. This structural organization contributes to the fibers`s mechanical robustness. While larger crystallite sizes are typically associated with reduced hydrophilicity and chemical reactivity, some studies have reported that increased crystallite dimensions can also enhance water uptake and reactivity due to the disruption of amorphous regions within the fiber matrix^19^. The favorable combination of crystallinity and crystallite size suggests that *Annona reticulata* fiber is a promising candidate for reinforcement in composite materials, particularly in applications requiring eco-friendly, biodegradable, and structurally resilient fibers.

Table 4 further supports this potential by comparing the crystallinity and thermal performance of *Annona reticulata* fiber with other natural fibers. It demonstrates superior crystallinity and competitive thermal performance, distinguishing it from alternatives such as *Ficus carica*, *Lygeum spartum*, and *Fishtail Palm Leaf Stalk*.

**Table 4:** Comparison of crystallinity and thermal stability of *Annona reticulata* fibers with other natural fibers.

Fibers	Crystallinity index (%)	crystallite size (nm)	Thermal stability (T_max_) (^o^C)	References
*Annona reticulata*	65	3.34	373	This work
*Cymbopogon nardus* root	59.16	2.13	368	^51^
Saharan aloe vera	52.6	5.6	350	^52^
*Ficus Carica* bark	41.42	5.51	333.68	^53^
*Lygeum spartum*	46.19	--	338.7	^54^
*Cayratia pedata*	67.84	2.71	335	^55^
Fishtail Palm *(Caryota mitis)* Leaf Stalk	45.70	16.92	326	^56^
*Abelmoschus fculneus*	56.25	4.17	349.83	^57^
Redish shell bean	57	3.9	328.23	^58^
Star jasmine	87.68	3.9	338.2	^28^
*Coccinia Grandis. L*	52.17	13.38	351.6	^16^
*Careya Arborea*	85.05	7.40	385	^59^
*Hierochloe odarata*	63.8	--	352.2	^60^

### Thermal stability analysis

**Fig. 4:**
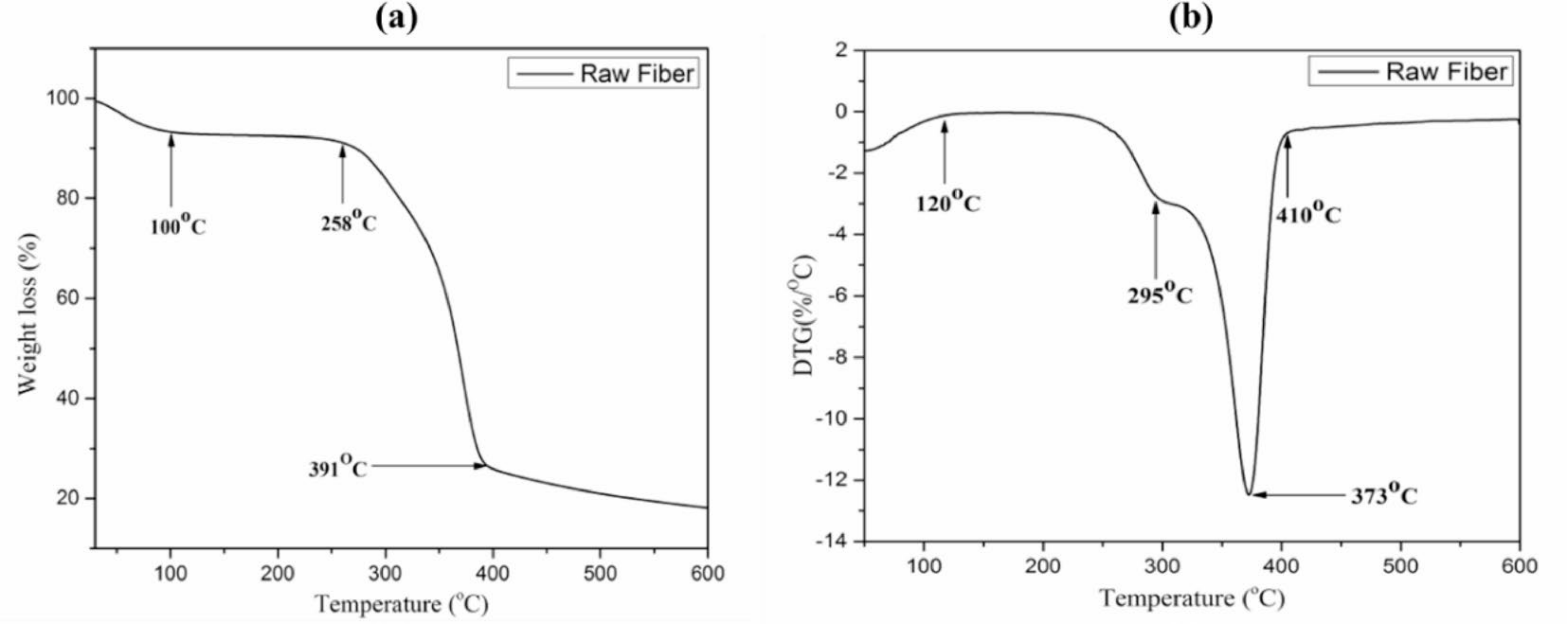
TG (**a**) and DTG (**b**) curves of *Annona reticulata* fiber.

The thermal degradation behavior of *Annona reticulata* fiber, evaluated through TGA and DTG is presented in Fig. 4, provides critical insight into its suitability for thermally demanding applications such as polymer matrix composites. An initial weight loss of approximately 2.5% around 100 °C is attributed to moisture evaporation, confirming the hydrophilic nature of the fiber^36,50^. The thermal stability of lignocellulosic fibers is primarily influenced by their three main components: cellulose, hemicellulose, and lignin. A major thermal decomposition began after 258 °C, primarily attributed to the breakdown of hemicellulose. This is followed by a next decomposition phase occurring between 258 and 391 °C, due to the thermal degradation of hemicellulose and cellulose^50^. The next stage (after 391 °C) is mainly involved for cellulose and lignin decomposition. The maximum decomposition temperature (DTGₘₐₓ) was observed at 373 °C, which was mainly due to the degradation nature of α-cellulose, indicating excellent thermal resilience. The onset temperature for major degradation is 295 °C, which is relatively high for untreated natural fibers. A small shoulder on the DTG curve around this temperature may indicate the presence of residual hemicellulose or heterogeneous molecular weights of cellulose^61^. The high thermal stability (T_max_ = 373 °C) either matches or exceeds that of several well-known natural fibers, including *Cymbopogon nardus* and *Ficus carica* (*see* Table 4), confirming *Annona reticulata*’s potential in thermal load-bearing composite systems. The moderate thermal stability of this natural fiber makes it ideal for preparing sustainable lightweight composites where polymer processing stays below 200 °C, like with polypropylene and epoxy^62^.

The char residue of the investigated fiber, as determined by TGA analysis, indicates its thermal stability, combustion behavior, and flame resistance properties. The fiber exhibited minimal weight loss toward the end of the test, retaining 18.5% char residue at 600 °C. The analysis result was comparable to that of several other reported fibers, as shown in Table 5.

**Table 5:** Comparison of Char residue of *Annona reticulata* bark fiber with other natural fibers.

Fibers	Char residue (%)	References
*Annona reticulata* bark	18.5	This study
Sugar cane	21	^63^
Bamboo	23.3	^63^
Coconut	23.6	^63^
*Erythrina variegata*	19.61	^64^
Saharan aloe vera	~ 20	^52^

### Surface analysis

**Fig. 5:**
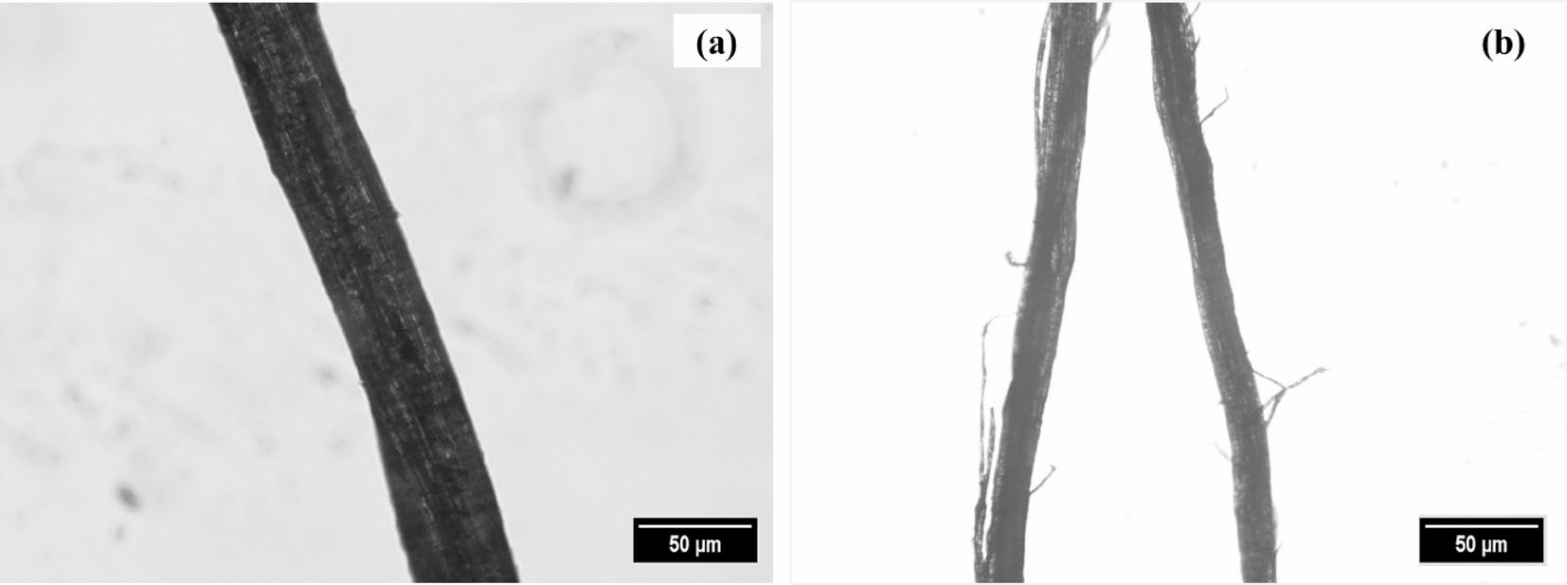
Microscopic view of fiber (**a**) 10× and (**b**) 4×.

The optical micromorphological images of *Annona reticulata* bark fibers are presented in Fig. 5, providing a preliminary view of their physical and structural characteristics. The raw fibers exhibit non-uniform diameters and relatively rough surfaces, with visible surface impurities. These features are primarily attributed to the bundle-type fiber structure and the presence of non-cellulosic components such as lignin and surface waxes^45,65^.

**Fig. 6:**
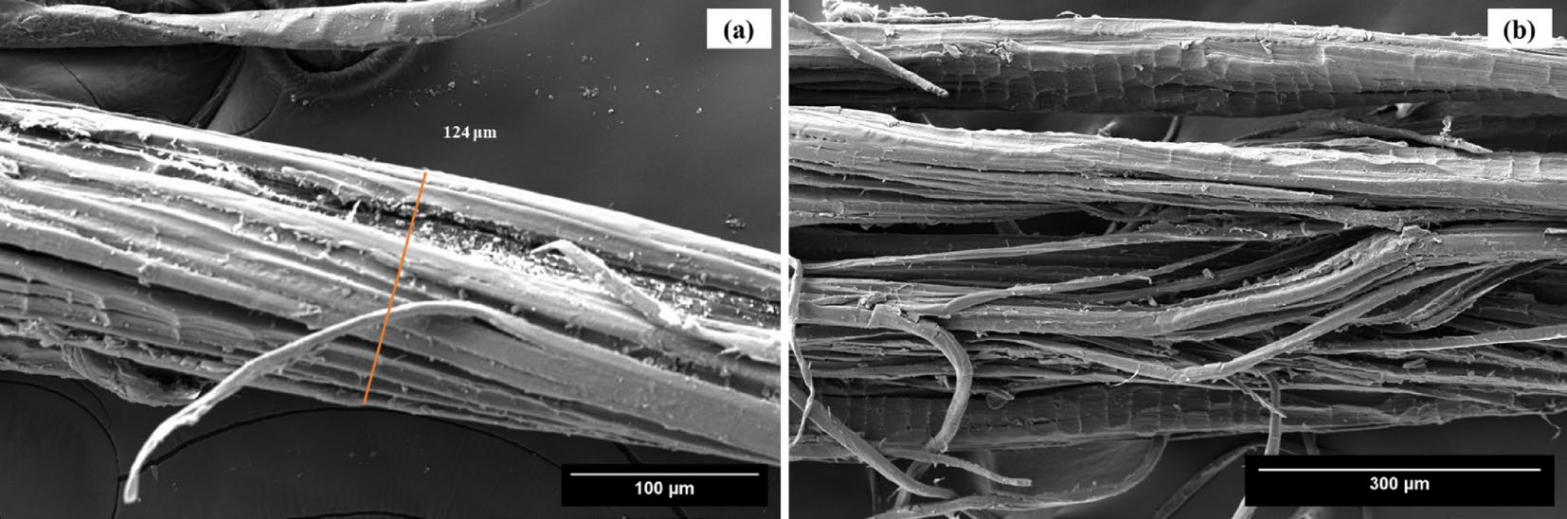
SEM micrographs of *Annona reticulata* fiber.

To gain deeper insight into fiber morphology, Scanning Electron Microscopy (SEM) was employed as presented in Fig. 6. The SEM images reveal irregular cross-sectional diameters (~ 124 μm) along with rough, and heterogeneous surfaces. Distinct surface flaws – including cracks, micro-voids, and helical fibrils are evident^18^. These microstructural features suggest a multi-fibrillar architecture, in which cellulose fibrils are bound together by non-cellulosic materials such as hemicellulose and lignin^65^. Additionally, the presence of waxes, oils, and other surface impurities is clearly visible, forming a compact alignment along the fiber axis. This compact yet impurity-laden structure is characteristics of raw lignocellulosic fibers^66^.

This surface morphology *Annona reticulata* fiber plays a critical role in determining its suitability for reinforcement in composite materials. Surface roughness enhances mechanical interlocking and promotes stronger fiber–matrix interfacial adhesion by increasing the effective surface area available for interaction with polymer matrices. These improved interfacial properties significantly influence load transfer efficiency and, consequently, the mechanical performance of the resulting composite. However, the presence of relatively smooth regions - attributed to waxy substances - may reduce wettability, indicating the potential need for surface treatments to optimize performance.

**Fig. 7:**
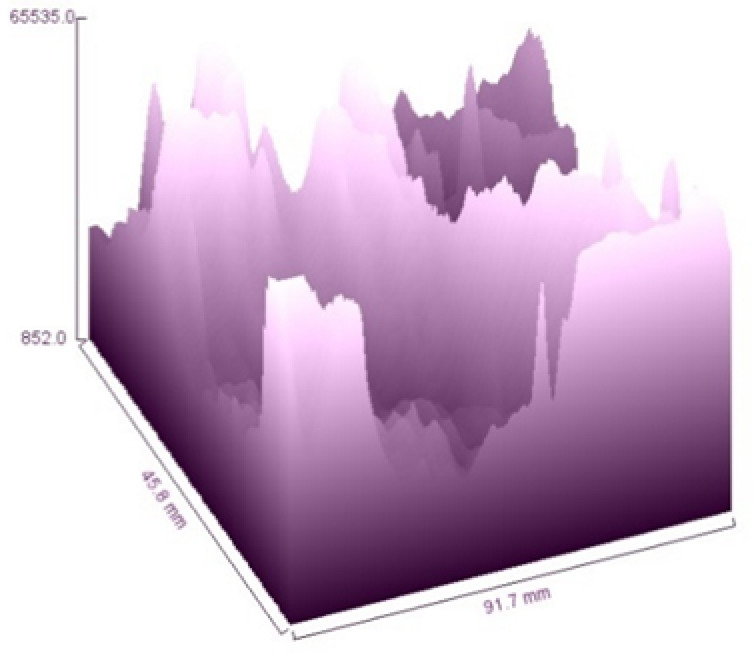
3D Surface roughness of *Annona reticulata* bark fibers.

The 3D surface roughness profile of *Annona reticulata* fiber, reconstructed from SEM data as shown in Fig. 7, offers quantitative insight into the topographical variance of the fiber surface^17^. Here, the x- and y-axes denote the pixel dimensions, while the z-axis represents grayscale-derived height, which correlates to electron emission intensity. The observed peaks and valleys illustrate the heterogeneous nature of the fiber’s surface topography.

This detailed surface topography is highly significant for multiple applications. For instance, in polymer composites, increased roughness enhances the interfacial bonding, while in textile applications, it improves dye uptake and coating adhesion^67^. Additionally, the presence of surface irregularities can enhance hydrophilicity, which is beneficial for functional finishing and the development of eco-friendly products.

### Physical and mechanical properties

The average diameter and length of *Annona reticulata* bark fibers were measured as 113–130 μm and approximately 5.5 cm, respectively. This fiber length is suitable for producing durable non-woven mats through carding and needle punching techniques. A fiber length of 1–3 mm is ideal for injection molding; however, length exceeding 5 mm may lead to machine clogging and poor fiber-matrix mixing dispersion. Nonetheless, longer fibers can still be utilized if processed into shorter lengths through nanofiber preparation methods such as acid hydrolysis or mechanical chopping. The fibers exhibit a bundled structure composed of numerous microfibrils held together by non-cellulosic components such as hemicellulose, lignin, and pectin.

Fiber density, a critical parameter in composite material selection, was recorded at 1.33 g/cm³. This relatively low density is advantageous for the development of lightweight composite materials, contributing to overall weight reduction without significantly compromising mechanical strength or stiffness. The measured density is slightly lower or closely comparable to other widely studied natural fibers like jute (1.3–1.49 g/cm³), kenaf (1.40 g/cm³), and flax (1.4–1.5 g/cm³)^52,68^.

Table 6 provides a comparative overview of the physical and mechanical properties of *Annona reticulata* fibers alongside other natural fibers. Notably, *Annona reticulata* exhibits a tensile strength of 134.5-522.7 MPa and a Young’s modulus of 1.6–6.8 GPa. These values fall within the performance range of several established fibers such as cotton, coir, and sisal^67,68^. The lower Young’s modulus value of this extracted fiber makes it more compatible with flexible matrices, impact-resistant application, or hybrid composite systems. Design framework should focus on optimizing interfacial adhesion and potentially blending with higher-modulus fibers to get excellent flexibility and strength performance.

Due to its acceptable specific strength and low density-comparable to other natural fibers as such as jute, *Hibiscus canescens*, cotton, coir, flax, and kenaf-this fiber appears suitable for the development of light weight composite materials. It holds strong potential to compete in applications such as transportation, consumer goods, and eco-friendly composite material production, offering a renewable alternative to high-density synthetic fibers with lower energy requirements during manufacturing.

**Fig. 8:**
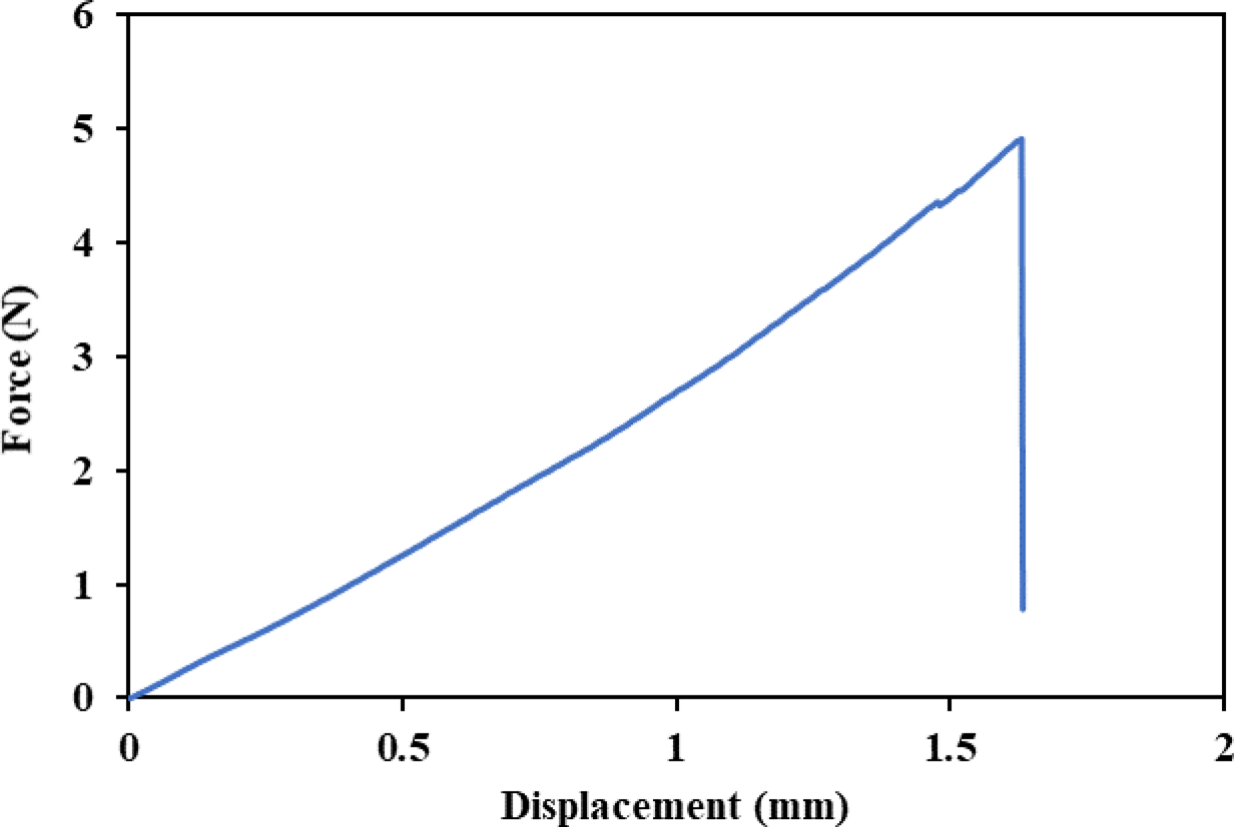
Tensile load-displacement curve of *Anonna reticulata* bark fiber.

Figure 8 illustrates the typical load vs. displacement behavior of *Annona reticulata* fibers. The curve exhibits an initial linear region corresponding to elastic deformation, followed by a gradual increase in strain until failure, reflecting the fiber’s inherent ductility and toughness.

Although the tensile strength and modulus values are moderate compared to high-strength natural fibers (e.g., flax and kenaf), the combination of adequate strength, low density (1.33 g/cm^3^), and moderate elongation at break (1-1.6%) offers a well-balanced set of mechanical properties. This balance is particularly advantageous for applications requiring both flexibility and toughness. Additionally, the observed mechanical performance highlights the potential of *Annona reticulata* fibers for use in moderate-load-bearing biocomposites, textile reinforcements, and other sustainable engineering applications.

**Table 6:** Comparison of mechanical properties of *Annona reticulata* bark fibers with other natural fibers.

Fiber	Fiber diameter (µm)	Density (g/cm^3^)	Tensile strength (MPa)	Tensile modulus (GPa)	Elongation at break (%)	Moisture content (%)	References
*Annona reticulata*	113–130	1.33	134.5-522.7	1.6–6.8	1-1.6	10.47	This work
Jute	20–200	1.3–1.49	320–800	8–78	1–1.8	12.5–13.7	^8^
Fishtail palm	380.5	1.47	249.23	--	2.01	12.19	^56^
Reddish shell bean	785.87	1.58	111	6.11	1.83	--	^58^
*Hibiscus canescens*	441.32	1.42	394.9	30.29	5.32	10.44	^68^
Bamboo	25–40	1.6–1.1	140–800	11–32	2.5–3.7	--	^8^
Cotton	10–45	1.5–1.6	287–800	5.5–12.6	3–10	7.85–8.5	^8^
Coir	10–460	1.15–1.46	95–230	2.8-6	15-51.4	8.0	^8^
*Ficus carica*	--	1.42	482 ± 6	--	2.72–4.91	9.07	^53^
Flax	12–600	1.4–1.5	343–2000	27.6–103	1.2–3.3	8–12	^8^
Kenaf	--	1.40	223–930	14.5–53	1.5–2.7	--	^8^
Sisal	8-200	1.33–1.50	363–700	9–38	2–7	10–22	^8^
S. aloe vera cactus leaves	91.15	1.32	621.8	40.03	2.47	7.6	^52^
*Hierochloe odarata*	136.71	1.16	105.73	2.56	2.37	--	^60^
*Lygeum spartum*	180–433	1.5	64.63-280.03	4.47–13.27	1.49–3.74	--	^54^

## Conclusions

This study presents a comprehensive investigation into the extraction, characterization, and evaluation of *Annona reticulata* bark fibers as a novel source of lignocellulosic fibers. Utilizing a short-duration chemical-free water retting process, cellulose-rich fibers were successfully isolated and systematically analyzed through physicochemical, morphological, thermal, and mechanical assessments. The key findings are summarized below:

- The fibers exhibited a high cellulose content (56.26 wt%), along with moderate levels of hemicellulose (17.56 wt%) and lignin (16.74 wt%), which aligns with the composition of many established natural fibers. This composition confirms their potential for functional applications. The elevated cellulose content contributes to improved mechanical properties such as strength and stiffness.
- FTIR spectroscopy confirmed the presence of characteristic functional groups associated with lignocellulosic biomass. In addition, SEM imaging revealed a fibrillar, bundled morphology, which is favorable for effective fiber–matrix interfacial bonding in composite systems. The prominent cellulose-related peaks reflect a well-organized fibrous network.
- The relatively low density (1.33 g/cm³) of the fiber compared to synthetic fibers contributes to a higher strength-to-weight ratio, making it advantageous for lightweight composite applications. Furthermore, the fiber exhibits an average tensile strength of 327 MPa, indicating its potential as a cost-effective alternative to glass or carbon fibers in various structural and functional applications.
- XRD analysis indicated a crystallinity index of 65% and a crystallite size of 3.34 nm, suggesting a moderately ordered cellulose structure. TGA results demonstrated thermal stability up to 258 °C, confirming the fiber’s compatibility with common polymer processing and reinforcing its potential for thermally stable composite applications.

This study is the first to explore the potential of *Annona reticulata* bark fibers as an underutilized biomass resource. The findings not only contribute to the growing database of biofibers but also highlight the value of converting biomass waste into high-performance, sustainable materials.

In summary, *Annona reticulata* bark fibers exhibit a well-balanced profile of mechanical, thermal, and structural characteristics, making them a strong candidate in the field of sustainable composite materials. Future research may focus on surface modifications, fiber-matrix interactions, and composite performance evaluation to further enhance the applicability of this novel fiber in advanced material systems.

## Data Availability

All data generated or analyzed during this study are included in this manuscript.
